# Effects of pistachios on body weight in Chinese subjects with metabolic syndrome

**DOI:** 10.1186/1475-2891-11-20

**Published:** 2012-04-03

**Authors:** Xin Wang, Zhaoping Li, Yanjun Liu, Xiaofeng Lv, Wenying Yang

**Affiliations:** 1 Department of Endocrinology, Sino-Japan Friendship Hospital, Beijing 100029, China; 2 David Geffen School of Medicine at UCLA, Los Angeles, USA; 3 the 306 Hospital of P.L.A, Beijing, China; 4 The Military General Hospital of Beijing, Beijing, China

## Abstract

**Background:**

Studies have shown that pistachios can improve blood lipid profiles in subjects with moderate hypercholesterolemia which could reduce the risk of cardiovascular disease. However, there is also a widely perceived view that eating nuts can lead to body weight gain due to their high fat content.

**Purpose:**

To investigate the impact of different dosages of pistachios on body weight, blood pressure, blood lipids, blood glucose and insulin in subjects with metabolic syndrome.

**Methods:**

Ninety subjects with metabolic syndrome (consistent with 2005 International Diabetes Federation metabolic syndrome standard without diabetes) were enrolled in three endocrinology outpatient clinics in Beijing. All subjects received dietary counseling according to the guidelines of the American Heart Association Step I diet. After a 4 week run-in, subjects were randomized to consume either the recommended daily serving of 42 g pistachios (RSG), a higher daily serving of 70 g pistachio (HSG) or no pistachios (DCG) for 12 weeks.

**Results:**

Subjects in all three groups were matched at baseline for BMI: DCG 28.03 ± 4.3; RSG 28.12 ± 3.22; and HSG 28.01 ± 4.51 kg/m^2^. There were no significant changes in body weight or BMI in any groups during the study nor any change from baseline at any time point in any group. During the entire study, there were no significant differences in waist-to-hip ratio among the groups or any change from baseline in any group (DCG -0.00 ± 0.03, RSG -0.01 ± 0.02 and HSG 0.01 ± 0.04). There were no significant differences detected among groups in triglycerides, fasting glucose and 2 hour postprandial glucose following a 75 gram glucose challenge. Exploratory analyses demonstrated that glucose values 2 h after a 75 gm glucose challenge were significantly lower at week 12 compared with baseline values in the HSG group (-1.13 ± 2.58 mmol/L, *p *= 0.02), and a similar trend was noted in the RSG group (-0.77 ± 2.07 mmol/L, *p *= 0.06), while no significant change was seen in the DCG group (-0.15 ± 2.27 mmol/L, *p *= 0.530). At the end of study, serum triglyceride levels were significantly lower compared with baseline in the RSG group (-0.38 ± 0.79 mmol/L, *p *= 0.018), but no significant changes were observed in the HSG or DCG groups.

**Conclusion:**

Despite concerns that pistachio nut consumption may promote weight gain, the daily ingestion of either 42 g or 70 g of pistachios for 12 weeks did not lead to weight gain or an increase in waist-to-hip ratio in Chinese subjects with metabolic syndrome. In addition, pistachio consumption may improve the risk factor associated with the metabolic syndrome.

## Introduction

Metabolic syndrome (MS) is a cluster of cardiovascular risk factors, including abdominal obesity, increased blood pressure, and blood glucose and lipid abnormalities often characterized as prediabetes. Obesity and metabolic syndrome are increasingly common in developing nations, including East Asian countries such as China, Japan, and Korea [1] and are associated with diabetes and increased cardiovascular disease mortality [2].

Epidemiological evidence has shown that frequent ingestion of nuts is beneficial in helping to reduce risk factors for coronary heart disease [3-5]. Increased consumption of pistachios has been shown to improve blood glucose level, endothelial function, and some indices of inflammation and oxidative status in healthy young men [6]. In subjects with moderate hypercholesterolemia pistachio consumption has been shown to improve blood lipids abnormalities [7]. Some of these beneficial effects may be related to the antioxidants naturally present in pistachio, since pistachio consumption has been shown to reduce oxidative stress markers in healthy subjects [8].

There is a widely held view that, due to its high fat content, nut consumption may lead to weight gain in the population at large especially in obese subjects and those with MS profiles. Li et al reported that the daily consumption of a defined quantity of pistachio nuts, when compared to an isocaloric refined carbohydrate snack, did not interfere with weight loss and improved triglyceride levels in an outpatient weight loss trial in the US [9]. However, there has not been a study of the impact of pistachio consumption on body weight and other risk factors for metabolic syndrome in Chinese patients. The present study evaluated the impact of daily consumption of no pistachios or two different amounts of pistachios on body weight gain, and various risk factors related to metabolic syndrome in Chinese subjects.

## Methods

### Subjects

The study protocol was approved by ethics committees at each of the participating hospitals. Written informed consent was obtained from the patient for publication of this report and any accompanying images.

Male and female subjects 25 to 65 years of age who also met the 2005 International Diabetes Federation (IDF) metabolic syndrome standards for a Chinese population [presence of three of the following risk factors of fasting serum triglyceride > 150 mg/dl, abdominal obesity (waist circumference > 90 cm in males or > 80 cm in females) or hypertension (SBP:≥130 or DBP ≥ 85 mmHg or history of treatment with anti-hypertensive agents) or impaired glucose tolerance (FPG > 100 mg/dl/or PPG > 140 and < 200 mg/dl) or low HDL-C (< 40 mg/dl in males or < 50 mg/dl in females)] and without diabetes were enrolled in three endocrinology outpatient clinics in Beijing, China. Subjects with type 2 diabetes, triglycerides > 500 mg/dL, LDL-C > 160 mg/dL, uncontrolled hypertension, hypothyroidism, Cushing's disease, polycystic ovarian syndrome, acute infectious disease or chronic diseases in liver, kidneys, heart, lungs or other organs were excluded.

All eligible subjects completed a four week run-in period to monitor the stability of vital signs, body weight and compliance. Subjects were given information on American Heart Association Steep 1 diet, not to consume any tree nuts. Any subjects with weight change ≥ 1 kg during the run-in was excluded from the study.

A randomization scheme was generated, and after the run-in period, subjects from each site were randomly assigned to one of the following three groups: recommended serving pistachio group (pistachios 42 g daily, RSG), high serving pistachio group (pistachios 70 g daily, HSG) and diet control group (DCG) with no pistachios Pistachio nuts were supplied in pre-portioned packets (Paramount Farms, Inc., Los Angeles, CA 90064) and were distributed to the subjects in intervention groups at each study visit at weeks 2, 4, 6,8,10 and 12. Subjects were instructed to consume pistachio as an afternoon snack.

All subjects were counseled on healthy eating at each visit according to the guidelines of the American Heart Association Step I diet. Subjects were asked not to consume any tree nuts other than those provided by the study. Subjects were instructed not to change their usual activity and exercise during the entire study period. A random phone call was conducted every 2 weeks for compliance of taking pistachio, following study instruction on diet and activity.

### Anthropometric measurements

The body weight and vital signs were measured at every study visit. Subjects were weighed at each visit on the same scale (RGZ-120-RT, Zeast Hangzhou, China) in light garments, barefoot and after an overnight fast. Height was measured with a stadiometer at week 0. BMI was calculated as weight (kg)/height squared (m). Waist circumference was measured midway between iliac crest and the lowermost margin of the rib. Hip circumference was measured at the maximum circumference of the buttocks with the subject standing with the feet placed together. Blood pressure was measured manually with subjects in a sitting position after resting > 10 minutes.

### Biochemistry

Blood samples were taken after 10-12 hours of fasting at baseline, and at weeks 6 and 12. Insulin was measured using an established radioimmunoassay in the endocrinology laboratory of China-Japan Friendship Hospital and the other indicators were measured in the clinical laboratories at each study location. Total cholesterol, triglycerides, and HDL were determined using standard enzymatic methods using a Roche Hitachi™ Analyzer and RANDOX reagents. LDL cholesterol was estimated from these data using the Friedewald equation. An oral glucose tolerance test (OGTT) was performed at baseline, and at weeks 6 and 12. A glucose challenge of 75 g was administered after a 10-12-hour fast, and blood glucose and insulin were measured at time 0, 1 hour and 2 hours. AST and ALT were measured with commercial analytical systems standardized to the International Federation of Clinical Chemistry and Laboratory Medicine (IFCC) recommended reference measurement systems using a Roche Hitachi™ Analyzer and RANDOX reagents.

### Data management and statistical analyses

All data management and statistical analyses were performed using SAS 9.13 software. Data were expressed as least squares mean ± standard deviation and checked for normality. The values reported were non-adjusted values. The difference of each indicator value before and after the experiment was measured by paired t test while the differences between groups were tested by analysis of variance. Confidence levels for significance were *P *< 0.05.

## Results

### Subjects

Ninety subjects who met the inclusion criteria were randomized to the study. The subject demographics and metabolic profiles are summarized in Table 1. The RSG group included 16 males and 14 females, with an average age of 51.89 ± 8.82 years, the HSG group included 12 males and 18 females, with an average age of 51.83 ± 9.37 years; and the DCG group included 13 males and 17 females, with an average age of 50.66 ± 9.86 years. There were no differences in the average age or gender distribution of the groups at the time of enrollment. There were no differences among the groups in mean body mass index (BMI), waist and hip circumference, fasting glucose, insulin or blood lipid levels (Table 1).

**Table 1:** Demographic and metabolic status of study subjects at baseline

Index	RSG (n = 30)	HSG (n = 30)	DCG (n = 30)
Age	51.89 ± 8.82	51.83 ± 9.37	50.66 ± 9.86

Gender	Male (53%)	Male (40%)	Male (43%)
	Female (47%)	Female (60%)	Female (57%)

BMI (kg/m^2^)	28.12 ± 3.22	28.01 ± 4.51	28.03 ± 4.35

Waist-to-hip ratio	0.93 ± 0.06	0.92 ± 0.06	0.92 ± 0.07

Fasting Glucose (mmol/L)	5.30 ± 0.85	5.18 ± 0.71	5.27 ± 0.79

Fasting Insulin (mU/L)	14.13 ± 6.89	17.08 ± 13.75	16.74 ± 14.81

Total cholesterol (mmol/L)	5.29 ± 1.01	5.29 ± 0.93	5.01 ± 0.99

Triglyceride (mmol/L)	2.47 ± 1.28	2.19 ± 0.76	3.11 ± 3.45

LDL(mmol/L)	3.08 ± 0.70	3.00 ± 0.70	2.70 ± 0.90

HDL (mmol/L)	1.15 ± 0.28	1.27 ± 0.30	1.05 ± 0.26

Analysis of variance was used for comparisons among the groups.

Of the 90 randomized subjects, 86 subjects completed the study. Four subjects dropped out of the study, two subjects in RSG group and one subject in the HSG group were lost to follow up, and one patient in RSG group withdrew from the study due to a diagnosis of diabetes that required treatment. There were no significant adverse events reported.

### Body weight and waist/hip ratio

**Figure 1:**
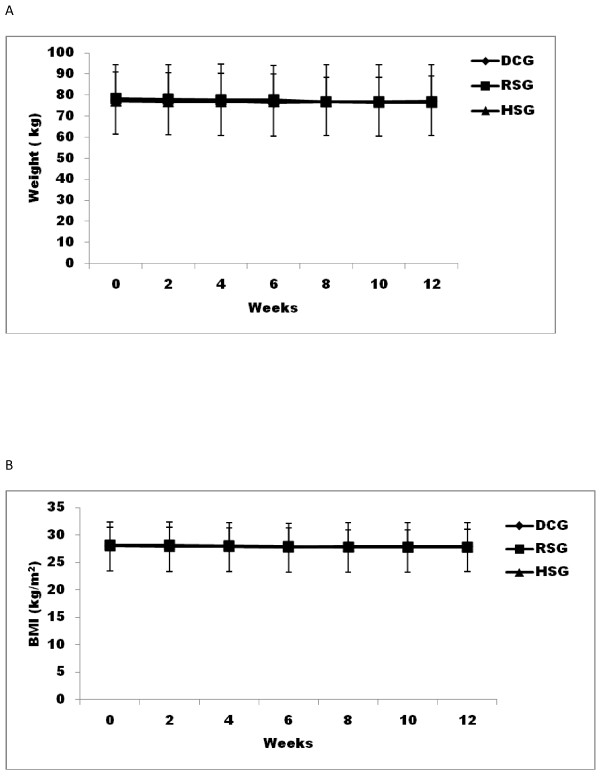
**Body weight (A) and BMI (B) in 12 weeks**. - ◆- dietary control group (DCG), -■-recommended serving of pistachio (RSG), -▲-high serving of pistachio (HSG). BMI was calculated as body weight in kilograms divided by the square of height in cm.

**Figure 2:**
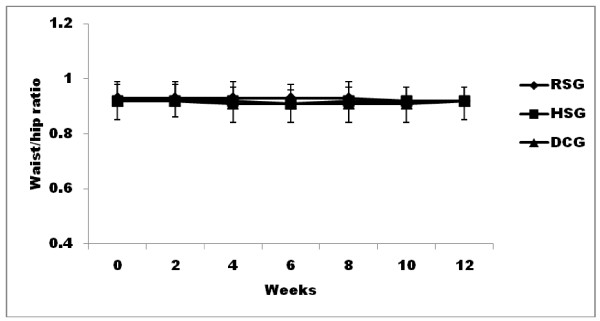
**Waist/hip ratio in 12 weeks**. -♦- dietary control group (DCG), -■- recommended serving of pistachio (RSG), -▲-high serving of pistachio (HSG).

The body weight at baseline was well matched among the three groups. There were no significant changes in mean body weight in any of the groups over the course of the study or at any study point. (Figure 1A). BMI at baseline in the three groups were 28.03 ± 4.3, 28.12 ± 3.22, and 28.01 ± 4.51 kg/m^2 ^in the DCG, RSG and HSG groups, respectively. There was no significant change in BMI from baseline at any time point in any of the groups. (Figure 1B). At Week 12, there were no significant changes from baseline in waist-to-hip ratio for the subjects in any groups (RSG - 0.01 ± 0.02, HSG 0.01 ± 0.04, DCG - 0.0 ± 0.03). (Figure 2)

### Blood pressure

**Figure 3:**
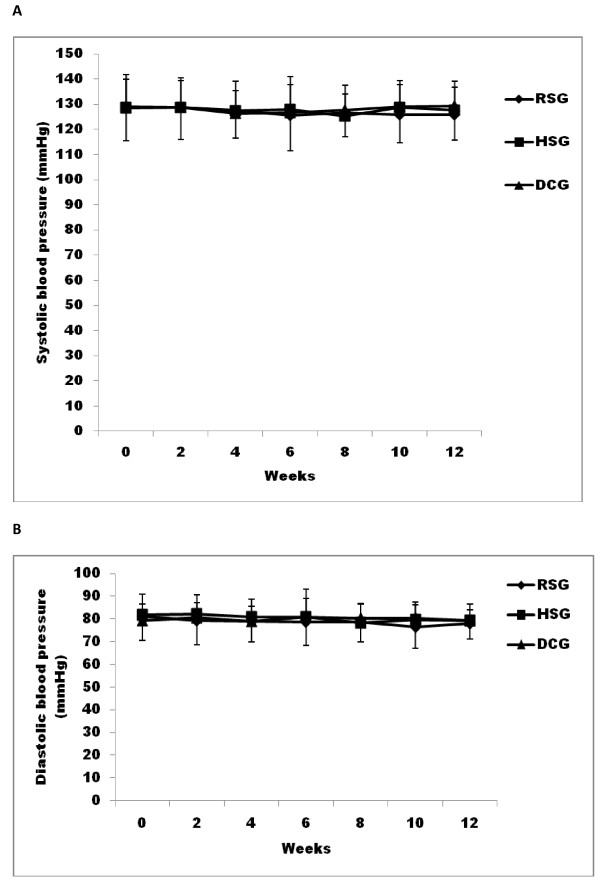
**Systolic (A) and diastolic (B) blood pressure in 12 weeks**. Systolic (A) and diastolic (B) blood pressure in 12 weeks. -**♦**- dietary control group (DCG), -■- recommended serving of pistachio (RSG), -▲-high serving of pistachio (HSG)

The baseline systolic and diastolic blood pressure were within a normal blood pressure range and without differences among the groups (systolic blood pressure was 129.1 ± 13.6, 128.7 ± 13.0, and 128.5 ± 11.4 mmHg for RSG, HSG, DCG groups; diastolic blood pressure was 81.1 ± 10.6, 81.7 ± 9.1, and 79.3 ± 7.3 mmHg for RSG, HSG, DCG groups, respectively)(Figure 3). There were no significant differences among the groups during the 12 week study period. In a secondary analysis the diastolic blood pressure in the RSG group at week 10 was lower than at baseline by 4.96 ± 10.1 mmHg (*p *= 0.017).

### Blood glucose and insulin levels

The fasting blood glucose levels at baseline were 5.30 ± 0.85, 5.18 ± 0.71, and 5.27 ± 0.79 mmol/L for RSG, HSG and DCG, respectively. There were no significant differences in values among the groups. Compared with baseline values, blood glucose increased significantly in the control group at week 12 (0.32 ± 0.65 mmol/L, *p *= 0.014) but not in the two pistachio groups (RSG 0.14 ± 0.68 mmol/L (*p *= 0.296); HSG 0.04 ± 0.74 mmol/L (*p *= 0.758). Blood glucose measured 2 hours after a 75 g glucose challenge were no statistical differences among the groups (*p *= 0.530) (Table 2) at baseline and end of the study. At week 12, the post challenge blood glucose decreased significantly from baseline in the HSG group (-1.13 mmol/L, *p *= 0.028) but there was no change in the DCG group (-0.15 mmol/L, *p *= 0.718) and only a non-significant downward trend in the RSG group (-0.77 mmol/L *p *= 0.064).

**Table 2:** Changes of blood glucose and insulin from baseline at week 12

Indicator	RSG (n = 27)		Change	HSG (n = 29)		Change	DCG (n = 30)		Change
	baseline	week 12		baseline	week 12		baseline	week 12	
GLU0h(mmol/L)	5.30 ± 0.85	5.41 ± 0.63	0.14 ± 0.68	5.18 ± 0.71	5.20 ± 0.60	0.04 ± 0.74	5.27 ± 0.79	5.57 ± 0.90	0.32 ± 0.65*
GLU2h(mmol/L)	8.16 ± 2.76	7.10 ± 2.08	- 0.77 ± 2.07	7.66 ± 2.08	6.66 ± 2.11	- 1.13 ± 2.58**	7.42 ± 2.00	7.27 ± 2.16	- 0.15 ± 2.27
Insulin 0h(mu/L)	14.13 ± 6.89	14.41 ± 8.68	0.47 ± 4.44	17.08 ± 13.75	14.68 ± 6.86	- 2.08 ± 14.54	16.74 ± 14.81	18.66 ± 22.48	1.93 ± 12.68
Insulin 2h(mu/L)	94.42 ± 78.42	83.50 ± 56.16	- 7.46 ± 50.52	83.80 ± 49.56	81.40 ± 42.14	- 11.10 ± 70.27	94.32 ± 84.18	81.99 ± 60.83	- 12.33 ± 55.59

t test was used for comparisons with baseline, * p < 0.05, ** p < 0.01.

### Blood lipids

There was no significant difference among the three groups in total cholesterol, triglycerides, LDL or HDL over the course of the study (Table 3). The triglycerides were within normal range at baseline (RSG 2.47 ± 1.28 mmol/L; HSG 2.19 ± 0.76 mmol/L; and DCG 2.09 ± 0.78 mmol/L). Triglyceride levels in the RSG group were lower at week 6 (-0.12 ± 1.06 mmol/L, *p *= 0.549) and decreased significantly from baseline by week 12 (-0.38 ± 0.79 mmol/L, *p *= 0.018). Compared with baseline values, the low density lipoprotein cholesterol (LDL) level was increased at week 12 for HSG (0.31 ± 0.51 mmol/L, *p *= 0.003) and DCG (0.29 ± 0.74 mmol/L, *p *= 0.037) but not for RSG (0.12 ± 0.50 mmol/L, *p *= 0.231). There was a 0.09 mmol/L decrease in LDL after 6 weeks in RSG compared to a 0.12 mmol/L increase in LDL for the DCG. There was no significant change in total cholesterol or high density cholesterol from baseline in any of the three groups.

**Table 3:** Change of blood lipids level

Parameter	RSG (n = 27)		Change	HSG (n = 29)		Change	DCG (n = 30)		Change
						
	baseline	week 12		baseline	week 12		baseline	week 12	
TC (mmol/L)	5.29 ± 1.01	5.20 ± 0.94	-0.01 ± 0.67	5.29 ± 0.93	5.41 ± 1.06	0.11 ± 0.69	5.01 ± 0.99	5.15 ± 1.04	0.14 ± 0.56
TG (mmol/L)	2.47 ± 1.28	2.14 ± 1.03	-0.38 ± 0.79*	2.19 ± 0.76	2.05 ± 0.80	-0.18 ± 0.87	2.09 ± 0.78	1.88 ± 0.89	-0.21 ± 0.53
LDL-C(mmol/L)	3.08 ± 0.70	3.10 ± 0.69	0.12 ± 0.50	3.00 ± 0.70	3.30 ± 0.78	0.31 ± 0.51**	2.70 ± 0.90	2.99 ± 0.86	0.29 ± 0.74*

t test was used for comparisons with baseline, * p < 0.05, ** p < 0.01.

### Liver function tests

Alanine transaminase (ALT) and Aspartate transaminase (AST) were measured. There was no significant change in ALT values during the study in any of the groups. The AST level remained at the same level for the control group but significantly decreased at week 12 for both pistachio groups (RSG - 7.81 ± 12.60 U/L, *p *= 0.003; HSG -5.52 ± 13.90 U/L, *p *= 0.041).(Table 4)

**Table 4:** Change of ALT and AST

	RSG (n = 27)		Change	HSG (n = 29)		Change	DCG (n = 30)		Change
	baseline	week 12		baseline	week 12		baseline	week 12	
Alanine transaminase (ALT), U/L	30.93 ± 16.10	30.12 ± 17.91	- 0.81 ± 8.97	28.20 ± 21.86	29.99 ± 18.48	1.79 ± 15.39	22.73 ± 13.43	29.06 ± 24.06	6.33 ± 14.43*
Aspartate transaminase (AST), U/L	28.93 ± 13.45	21.11 ± 7.60	-7.81 ± 12.60**	28.50 ± 13.24	22.98 ± 8.85	-5.52 ± 13.90*	23.13 ± 6.81	23.47 ± 12.5	0.34 ± 10.68

t test was used for comparisons with baseline, * p < 0.05, ** p < 0.01.

## Discussion

Studies have shown that nut consumption is of benefit in the prevention of cardiovascular disease [10]. In a health claim approved by US FDA in July 2003, it was stated that "Scientific evidence suggests, but does not prove, that eating 1.5 ounces per day of most nuts, such as pistachio, as part of a diet low in saturated fat and cholesterol, may reduce the risk of heart disease."

There has been concern that consumption of pistachios may lead to increased calorie intake and body weight gain due to its fat content. If true, this finding would suggest that patients with metabolic syndrome should limit their intake of pistachios. Consumption of excess calories is of particular concern in Chinese individuals who have an overall lower BMI and basal metabolic rate compared to Western populations [11-13]. Our results indicate that the daily consumption of either a high dose or recommended dose of pistachio nuts for 12 weeks, when compared with a control group, resulted in no changes in body mass index or waist-to-hip ratio. These results were consistent with the results of previous studies in other populations [7,14]. In a 12 week parallel study of 118 subjects in which nuts were compared to other energy-dense snacks, nuts improved diet quality without any negative impact on body weight or lipid values [15]. The study conducted by Coates and Howe [16] indicated that nuts may be helpful for the regulation of body weight by inhibiting appetite and fat absorption. Studies have suggested that the lipid in nuts is more poorly absorbed than from other food sources. Baer et al reported that the measured energy density of pistachios was 22.6 kJ/g, which is 5% less than the currently accepted energy value of 23.7 kJ/g, as calculated using the Atwater general factors [17].

Pistachios have a low glycemic index. The study conducted by Josse [18] indicated that, when taken together with high carbohydrate foods, pistachio nuts decreased the absorption of carbohydrate, and lowered postprandial blood glucose. In the present study the primary analysis did not show any difference among groups but the secondary analysis did demonstrate that consuming 70 gram of pistachio nuts significantly lowered 2 hour blood glucose values after a 75 gram glucose challenge in subjects with metabolic syndrome.

It has been shown that nuts are not only beneficial in improving the blood lipid profile, but may also prevent coronary heart disease through the other mechanisms [5]. Hypertension is one of the criteria of metabolic syndrome. In this study the consumption of pistachios did not lead to increased blood pressure in patients with metabolic syndrome, consistent with prior study results [7,14].

Aminotransferase levels in obesity are associated with insulin resistance and the metabolic syndrome [19,20]. The AST level in our study was significantly lowered by consume pistachio. Pistachio nuts are rich in monounsaturated fatty acids, antioxidants such as vitamin E, lutein, b-carotene, and proanthocyanidins. Those nutrients have antioxidant properties [8,21], and may offer protection against oxidative stress in liver.

The purpose of the study was to demonstrate that pistachios do not cause weight gain predicted from their fat content in a free living population. The dietary intake and activity level were not controlled that was the limitation of current study. Although our secondary analysis comparing the results in the individual arms to baseline showed changes of some of the risk factors in the pistachio groups the study was underpowered to determine the differences among the groups and should be repeated in a larger population.

## Conclusion

Despite concerns that pistachio nut consumption may promote weight gain, the daily ingestion of either 42 g or 70 g of pistachios for 12 weeks does not lead to weight gain or an increase in waist-to-hip ratio in Chinese subjects with metabolic syndrome. In addition, pistachio consumption may also improve risk factors associated with the metabolic syndrome.

## Competing interests

This study was funded by a grant from Paramount Farms, Inc., Los Angeles, CA 90064. The authors declare that they have no other competing interests.

## Authors' contributions

XW has contributed to conduction of the study, acquisition of data, analysis and interpretation of data and has been involved in drafting the manuscript. WY and ZL have contributed to study conception and design, acquisition of data, analysis and interpretation of data and have been involved in drafting the manuscript. YL and XL have contributed to the conduction of the study and acquisition of data. All authors read and approved the final manuscript.
